# Basal ganglia modulation of thalamocortical relay in Parkinson's disease and dystonia

**DOI:** 10.3389/fncom.2013.00124

**Published:** 2013-09-05

**Authors:** Yixin Guo, Choongseok Park, Robert M. Worth, Leonid L. Rubchinsky

**Affiliations:** ^1^Department of Mathematics, Drexel UniversityPhiladelphia, PA, USA; ^2^Department of Mathematical Sciences and Center for Mathematical Biosciences, Indiana University Purdue University IndianapolisIndianapolis, IN, USA; ^3^Department of Neurological Surgery, Indiana University School of MedicineIndianapolis, IN, USA; ^4^Stark Neurosciences Research Institute, Indiana University School of MedicineIndianapolis, IN, USA

**Keywords:** thalamocortical relay, Parkinson's disease, dystonia, basal ganglia, globus pallidus

## Abstract

Basal ganglia dysfunction has being implied in both Parkinson's disease and dystonia. While these disorders probably involve different cellular and circuit pathologies within and beyond basal ganglia, there may be some shared neurophysiological pathways. For example, pallidotomy and pallidal Deep Brain Stimulation (DBS) are used in symptomatic treatment of both disorders. Both conditions are marked by alterations of rhythmicity of neural activity throughout basal ganglia-thalamocortical circuits. Increased synchronized oscillatory activity in beta band is characteristic of Parkinson's disease, while different frequency bands, theta and alpha, are involved in dystonia. We compare the effect of the activity of GPi, the output nuclei of the basal ganglia, on information processing in the downstream neural circuits of thalamus in Parkinson's disease and dystonia. We use a data-driven computational approach, a computational model of the thalamocortical (TC) cell modulated by experimentally recorded data, to study the differences and similarities of thalamic dynamics in dystonia and Parkinson's disease. Our analysis shows no substantial differences in TC relay between the two conditions. Our results suggest that, similar to Parkinson's disease, a disruption of thalamic processing could also be involved in dystonia. Moreover, the degree to which TC relay fidelity is impaired is approximately the same in both conditions. While Parkinson's disease and dystonia may have different pathologies and differ in the oscillatory content of neural discharge, our results suggest that the effect of patterning of pallidal discharge is similar in both conditions. Furthermore, these results suggest that the mechanisms of GPi DBS in dystonia may involve improvement of TC relay fidelity.

## Introduction

Dystonia is a widespread neurological disorder characterized by sustained muscle contractions, involuntary repetitive movements and abnormal posture. Although the exact pathophysiological mechanism remains unknown, prior studies have demonstrated involvement of basal ganglia circuitry (Fahn et al., 1998; Tarsy and Simon, 2006). Some dystonia cases have clear genetic origin, but there is no apparent neurodegeneration, although some functional and microstructural brain abnormalities have being detected and abnormalities of neural plasticity in sensorimotor networks have been described (Breakefield et al., 2008).

Another disorder where pathology of the basal ganglia is involved is Parkinson's disease. It is of interest to note that similar surgical procedures—pallidotomy (therapeutic lesion) and high-frequency electric deep brain stimulation (DBS) in the internal Globus Pallidus (GPi)—are used to treat the symptoms of both disorders [for example, see (Lozano et al., 1997) for pallidotomy in dystonia; (Vidailhet et al., 2005, 2007; Kupsch et al., 2006) for GPi DBS in dystonia; (Laitinen et al., 1992; Lang et al., 1997) for pallidotomy in Parkinson's disease; (Moro et al., 2010) for GPi DBS in Parkinson's disease]. Hence, comparison of basal ganglia activity in dystonia and Parkinson's disease may shed light on the pathophysiology of both disorders.

Firing rates in GPi—the output nuclei of basal ganglia—have been studied in detail in both conditions. While at least one study suggested that the firing rates are similar in both disorders (Hutchison et al., 2003), most studies suggest that pallidal firing rates in dystonia are lower than those in Parkinson's disease, which in turn, are lower than normal rates, as inferred from primate studies (Lenz et al., 1999; Merello et al., 2004; Starr et al., 2005; Tang et al., 2007), although it is less clear how much lower the rates are as the intraoperative mapping of neurons may not be very representative and may depend on a particular subtype of the disease (Tang et al., 2007). However, not only average firing rate but the temporal patterns of activity in basal ganglia circuits are related to symptomatology in both disorders, perhaps more than the average firing rates.

In Parkinson's disease (and experimentally created low-dopamine states), hypokinetic symptoms are associated with an increase of synchronized oscillatory activity in the β frequency band (10–30 Hz, broadly speaking) in basal ganglia-thalamocortical circuits including GPi (Hutchison et al., 2004; Brown, 2007; Hammond et al., 2007). Successful treatment of hypokinetic symptoms is marked by a decrease in this activity. While it has not being fully confirmed that this activity causes the symptoms, it is certainly closely related to their presence in Parkinson's disease (Eusebio and Brown, 2009).

Dystonia is also marked by changes in oscillatory activity, however, it appears to be in a different frequency band. Local field potentials (LFP's) (Silberstein et al., 2003; Liu et al., 2006) and unit discharges (Starr et al., 2005) in dystonia present increased oscillatory synchronized activity in low frequency bands, i.e., theta and alpha (roughly 3–12 Hz). LFP and spikes exhibit synchrony in this frequency range in dystonia (Chen et al., 2006) and the LFP is correlated with the electromyogram (EMG) (Sharott et al., 2008). Dystonic muscle spasms are preceded by an increase of the oscillatory power in low frequencies (Liu et al., 2008). Similar to Parkinson's disease the pathological oscillatory activity is observed in multiple parts of the basal ganglia-thalamocortical circuits in dystonia (Starr et al., 2005; Schrock et al., 2009). Animal models of dystonia exhibit similar low-frequency bursting too (Gernert et al., 2002; Chiken et al., 2008).

Disorganized patterns of activity in GPi (the output nucleus of basal ganglia) may disorganize the activity of downstream circuits, in particular, the dynamics of thalamocortical relay. GPi projects to thalamus and the presence of pathological oscillations in Parkinson's disease has being considered as a source of pathologically erratic thalamocortical relay, which was conjectured to contribute to the generation of Parkinsonian symptoms (Obeso et al., 2000, 2008; Rubin and Terman, 2004; Guo et al., 2008). In particular, utilization of data obtained from MPTP monkeys in a computational model of thalamocortical relay revealed how the presence of oscillations in basal ganglia activity modulates thalamocortical relay cells in such a way that they enter a more bursty mode, where they are less responsive to the incoming excitatory input (Guo et al., 2008).

The presence of altered oscillatory activity in dystonia suggests that it may affect the efficiency of thalamocortical relay and thus contribute to symptoms in this condition as well. This is the subject of the present study. In particular we explore the differences and similarities between parkinsonian and dystonic thalamocortical relay. The diseases are generally symptomatically distinguishable, have different etiologies, and are noted for difference in pathological neural activity. However, different pathological rhythmicities may have a similar impact on the downstream circuits and produce similar effects.

To study this problem we used a data-driven computational model: a computational model of thalamo-cortical relay modulated by real data recorded in GPi of parkinsonian and dystonic patients. The use of the real pallidal recordings in the computational model may be a substantial advantage. The temporal patterns of synchronous oscillations in PD are known to have a complicated structure (Park et al., 2010). Dystonia may also be marked by a similarly complicated temporal patterning of neuronal discharge. Thus, using the real data will allow us to capture the response of thalamocortical relay not only to bursting in a specified frequency range, but to real pallidal activity with its specific complex temporal structure.

## Methods

### Patients and surgery

This study included 4 patients with Parkinson's disease (2 males; ages, 52, 62, 67, 72 years; disease duration, 10, 14, 15, 18 years) and three patients with generalized dystonia (2 males; ages, 50, 55, 76 years; disease duration, 3, 11, 10 years), who underwent stereotactic procedures in GPi (pallidotomy or pallidal DBS) in Indiana University Hospital. The decision to perform the surgery was not influenced by subsequent inclusion of the data in the present study (which included all appropriate patients for whom the recordings were available). The surgical procedure was described in detail in (Schiff et al., 2002). Briefly, targeting was carried out using the Leksell frame and MRI scan with standard stereotactic coordinates for postero-ventral GPi. Localization was confirmed with postoperative MRI. At the time of surgery, patients had been off antiparkinsonian medication for at least 12 h. The protocol was approved by Indiana University IRB.

### Data recording and processing

Intraoperative recordings were performed with 80% platinum/20% iridium glass-insulated microelectrodes (FHC, Bowdoin, ME), with an impedance, measured in the brain at 1 kHz, being in the range of 0.3–1.0 MΩ. The recordings were made with Guideline System 3000 (Axon Instruments, Foster City, CA). The signal was amplified (×5000) and filtered to 300–5 kHz frequency bands to obtain the spiking neuronal unit signal, which was digitized at 20 kHz rate and saved for off-line analysis. Neuronal spikes were subsequently threshold extracted (>3 SD above baseline), single units were isolated with SciWorks/Experimenter software (DataWave Technologies, Berthoud, CO), and the time of occurrence of spikes was recorded and used to drive the thalamocortical relay model described below.

### Mathematical model of TC relay

We used the same computational model as the previous study on monkey data (Guo et al., 2008), which is a modified version of the TC model from (Sohal et al., 2000). In the TC model, the current-balance and ionic activation equations take the form

(1) $$\begin{array}{l} C_{m}dv/dt=-I_{L}-I_{Na}-I_{K}-I_{T}-I_{Gi\to Th}-I_{E}+I_{ext}\\ \;\frac{dh}{dt}=\frac{h_{\infty }\left(v\right)-h}{\tau_{h}\left(v\right)}\\ \;dr/dt=(r_{\infty }(v)-r)/\tau_{r}(v). \end{array}$$

In system (1), the terms *I*_*L*_ = *g*_*L*_(*v* − *E_L_*), *I*_*Na*_ = *g_Na_m*^3^_∞_(*v*)*h*(*v* − *E_Na_*), are leak and sodium spiking currents, respectively. The expression for the potassium current is *I*_*K*_ = *g_K_*(0.75(1 − *h*))^4^(*v* − *E_K_*). The low-threshold calcium current is *I*_*T*_ = *g*_*T*_*p*^2^_∞_(*v*)*r*(*v* − *E_T_*). For the intrinsic currents, the forms of the functions and the values of the parameters used appear in Table 1. The resting potential, spike threshold, and responsiveness of the model TC cell, in the absence of inputs, are robust to changes of ionic conductance in this model. The capacitance is *C*_*m*_ = 1 μF/cm^2^ after rescaling the parameters. The reversal potentials are given in mV, conductance in mS/cm^2^, and time constants in ms.

**Table 1:** **Functions and parameters for the TC cell**.

**Current**	**Activation**	**Inactivation**
*I*_*Na*_	*m*_∞_ (*v*) = 1/(1 + *e*^−(*v*+37)/7)^	*h*_∞_(*v*) = 1/(1 + *e*^(*v*+41)/4^)
	*τh (v)* = 1/ (*a_h_* (*v*) + *b_h_* (*v*)), *a_h_ (v)* = 0.128*e*^−(46+*v*)/18^, *b_h_(v)* = 4/(1 + *e*^−(23+*v*)/5)^	
*I*_*T*_	*p*_∞_ (*v*) = 1 / (1+ *e*^−(*v*+60)/6.2^)	*r*_∞_ (*v*) = 1 / (1 + *e*^(*v*+84)/4^)
	*τ_r_ (v)* = 0.4 (28 + *e*^−(*v*+25)/10.5^)
Parameters	*g_L_* = 0.05, *g_Na_* = 3, *g_K_* = 5, *g_T_* = 2, *v_L_* = −72, *v_Na_* = 50, *v_K_* = −90, *v_T_* = 90	

Additional terms in (1) refer to inputs to the TC cell model. The equations and parameter values relevant to these terms are summarized in Table 2, with the same units used as in Table 1.

**Table 2:** **Inputs to the TC cell**.

GPi synaptic input	*I_Gi_* → *Th* = *g_syn_* *s*(*v* − *E_syn_*), *s* = 1, at each spike, decays as *ds/dt* = −β_*inh*_ *s after each spike*	
Excitatory signal	I_*E*_ = *g_E_s_E_* (*v* − *v_E_*), where *s*_*E*_^'^ = (1 − *s_E_*)*exc(t)* − β*s_E_*	
	Periodic *exc* (*t*)	Random *exc* (*t*)
	*exc*(*t*) = 1 for *d* ms when excitatory signal is on;	*exc*(*t*) = 1 for *d* ms when excitatory signal is on;
	*exc*(*t*) = 0 in between excitatory signals	*exc*(*t*) = 0 in between excitatory signals. The onset of each signal is generated by modified Poisson processes with rate 0.005. The minimal interval between two signals is 20 ms.
Constant input		*I*_*ext*_ = 0.41
Parameters	*g*_*E*_ = 0.0 4, *g*_*syn*_ = 0.08, *v*_*E*_ = 0, *v*_*syn*_ = −85, β_*inh*_ = 0.1, α = 0.8, β = 0.25, *p* = 50 ms, *d* = 5 ms, *win*_*off*_ = 10 ms.	

*I*_*ext*_ = 0.41 is a fixed constant background input that yields a firing rate of roughly 12 Hz in the absence of excitatory signals and GPi inhibitory synaptic input. This value places the TC cell near the transition from silent to spontaneously oscillatory for the model TC cell, in the absence of all other inputs. *I*_*Gi* → *Th*_ represents the inhibitory input current from the GPi to the model TC cell. *I*_*E*_ denotes simulated periodic or stochastic excitatory input to the TC cell. It may represent synchronized inputs from multiple presynaptic cells. Earlier studies of this kind of TC modeling (Guo et al., 2008) and (Guo and Rubin, 2011) performed sensitivity analysis on the TC cell parameters with various (experimentally recorded and computer-generated) pallidal inhibition time-series. The results were very robust and small variation of several parameters of TC case in this study did not lead to different results.

### Excitatory signals

In the TC model, the excitatory signal *I*_*E*_ represents a set of temporally proximal, but imperfectly aligned cortical inputs to a TC cell. The excitatory input is modeled by *I*_*E*_ = *g_E_s_E_*(*v* − *v_E_*)and *s*_*E*_ is governed by

(2) $$ds_{E}/dt=\alpha \left(1-s_{E}\right)exc\left(t\right)\;-\;\beta s_{E},$$

where α = 0.8, and β = 0.25 as given in Table 2. Since we do not have the voltage signal of a presynaptic neuron in the model, we use the function *exc*(*t*) to control whether the excitatory input is on or off. Specifically, *exc*(*t*) = 1, during each excitatory input, while *exc*(*t*) = 0 between excitatory inputs. We used two general forms of time course for *exc*(*t*), namely periodic and stochastic, as done in the past work (Rubin and Terman, 2004; Guo et al., 2008).

In the periodic case, *exc*(*t*) = 1 from time 0 up to time *d*, from time *p* up to time *p* + *d*, from time 2*p* up to time 2*p* + *d*, and so on. We choose 50 ms for *p* so that the model TC cells rarely fire spontaneous spikes between excitatory signals of 20 Hz frequency. This frequency is also consistent with the high-pass filtered corticothalamic inputs observed *in vivo* (Castro-Alamancos and Calcagnotto, 2001). In the stochastic case, input onset times are selected from a modified Poisson distribution (Poisson distribution with an enforced pause of 20 ms between spikes to avoid excessive firing) with the same input duration and amplitude as in the periodic case and with a mean input frequency of 20 Hz. The use of a stochastic excitatory input provides both biological realism and one measure of the robustness of our results to noise. We select the values of the rate parameters α, β, and *d* based on corticothalamic excitatory inputs recorded *in vivo* (Castro-Alamancos and Calcagnotto, 2001). We choose the value for *g*_*E*_ based on two considerations. First, *I*_*E*_ should be strong enough to overcome the spontaneous oscillation in the model TC cell and respond faithfully to both the periodic and stochastic excitatory signals in the absence of GPi inhibitory input. Secondly, the choice of *g*_*E*_ was motivated by the conjecture that strong inputs would represent important signals, and that differences in TC relay of strong inputs would have the most significant impact on downstream processing.

### Inhibitory GPi input derived from patients' data

We model the inhibitory input as *I*_*Gi* → *Th*_ = *g_syn_ s*(*v* − *E_syn_*), *s* = 1, at each spike time of GPi recording, then decays according to

(3) $$\frac{ds}{dt}=-\beta_{inh}s.$$

In this study, each TC cell receives one GPi input *s* that is derived from spike trains recorded from either PD or DY patients. Spike sorting was performed to recover single units in off-line analysis (See section Data Recording and Processing).

### Evaluation of TC relay fidelity

The TC neuron responds with a single spike to some of the excitatory inputs that it receives, which is a high-fidelity response. The TC neuron may also produce either no spike or multiple spikes to one excitatory input under the GPi inhibition, which we consider as miss and bad responses, respectively.

In this paper, we quantify TC relay performance using two index numbers, one is the miss index, the other is the bad index. And they are defined as the following:

miss index = $\frac{m}{n}$, bad index = $\frac{b}{n}$,

where *b* denotes the number of excitatory inputs to which a TC neuron gives a bad response consisting of more than one spike, either a burst response (typically) or a single-spike response followed after a delay, but before the next input, by additional spikes. The number *m* denotes the count of excitatory inputs that are missed by the TC neuron, in the sense that it fails to fire any spikes during a detection window. The number *n* is the total number of excitatory inputs. The detection window we use in this paper extends from the beginning of each excitatory input to 15 ms after each input. For each excitatory input, either a bad or a miss TC response is counted if a faithful TC response does not occur during this window. The detection algorithm for both miss and bad responses was first implemented in Guo et al. (2008). In previous work a single error index that is the sum of miss and bad index was first introduced in (Rubin and Terman, 2004). Guo et al. (2008) also used a single error index as in Rubin and Terman (2004) to quantify how different patterns of inhibitory GPi signals obtained from experimental recordings of normal and parkinsonian monkeys, with and without DBS, affect TC relay responses. In this current work, we separate the TC relay error into the miss and bad indices to provide a better characterization of TC relay.

### GPi elevated spiking time and contributed silent time

We designed detection algorithms to identify the elevated spiking and contributed silent episodes in GPi. These episodes contribute to the miss index and bad index in TC cells described in the previous section. To quantify elevated spiking episodes (ESE), we use the same detection algorithm for high-frequency spiking episodes as was done in (Guo et al., 2008) with parameter adjustments. In this algorithm, we first detect all GPi spikes preceded by a silent period longer than 26 ms. We choose an interval of 26 ms to identify the potential start of ESEs because it is large enough so that we can detect the long interval between spikes, but not so large so that we will falsely identify bursts by including lots of isolated GPi spikes into a burst. If this number is too small, the algorithm could incorrectly separate one ESE into several short ESEs, with each short ESE only containing a couple of spikes. We chose an interval size that reduces these erroneous values. However, since our data sets are from real recordings of human patients, we cannot completely eliminate those two extremes. Nevertheless, we can monitor the ESE algorithm so that these unreasonable extremes rarely occur.

Using alternative values in the neighborhood of 26 ms does not affect the overall results of the indices. Each GPi spike with a proceeding silent period longer than 26 is a potential candidate for the start of an ESE. Such a GPi spike is considered as a start of an ESE only if the immediate next spike follows within 20 ms. Otherwise, it is considered as an isolated spike. All subsequent spikes thereafter are counted as part of the same episode if and only if they happen within 20 ms of their predecessors. Once a spike occurs beyond the 20 ms window, the preceding spike marks the end of the episode. The 20 ms maximum between spikes within each burst prevents us from falsely including isolated spikes in an ESE. Other spikes occurring in between ESE's are considered as random spontaneous spikes. We quantify ESEs by their duration from the first spike to the last spike. The fraction of ESE over the total simulation time is called EST (elevated spike time) and is a number between 0 and 1, with 0 EST corresponding to no bursting GPi episodes with only random isolated spikes. A moderate EST means a bursty GPi signal which is generally the case with our GPi signals since they are recorded from PD and DY patients.

In this work, we associate a new measure, contributed silent episode (CSE), with TC relay errors. We introduce this new measure in addition to EST used in previous work (Guo et al., 2008) for three reasons. First, the bad relay responses with excessive TC spikes to a single excitatory input usually happen during the silent time after ESEs due to post-inhibitory rebound. Secondly, the late part of a long silent episode in between two ESEs does not contribute to either miss or bad TC responses because post-inhibitory rebound only lasts for a brief duration of time after GPI inhibition. Third, when the silent episode between two ESEs is too short, is it not likely to have a bad TC response because post-inhibitory rebound has some latency.

The properties of post-inhibitory rebound, such as the latency of the first TC spike and the duration of post-inhibitory rebound after inhibition, depends on the neuron's intrinsic membrane currents and the amplitude and duration of the inhibition (Perkel and Mulloney, 1974; Hartline and Gassie, 1979; Harris-Warrick et al., 1995a,b; Hooper, 1998; Winograd et al., 2008). Throughout our simulation, all the intrinsic membrane currents of the model TC neuron and the amplitude of GPi inhibition are fixed. We assume that both factors, the intrinsic membrane currents and inhibition amplitude, have the same influence over all simulations in our study. Thus, we solely consider the effect of duration of GPi inhibition on the latency and duration of post-inhibitory rebound of the TC neuron.

We first find the potential non-bursting silent episodes by marking the time between the last GPi spike of an ESE and the first GPi spike in the next ESE. There are two cases for these non-bursting silent GPi episodes. Either there are no isolated GPi spikes during the interval or, only a few isolated GPi spikes occur. When the silent period (with or without isolated GPi spikes) between two ESEs is smaller than 35 ms, the time interval is too short for the TC cell to generate a bad response due to post-inhibitory rebound before the next GPi burst. We do not count these as CSEs. In the case when there are no isolated GPi spikes during a non-bursting silent period that is longer than 35 ms, we only use the beginning 100 ms as CSE because the proceeding GPi burst is unlikely to cause a bad TC response after 100 ms and before the next GPi burst. In the non-bursting episodes during which there are a few isolated GPi spikes, we look at the inter-spike intervals of the first three GPi spikes. For those spikes whose inter-spike interval is less 35 ms, they contribute little to the TC relay response. For those that are larger than 35 ms, we only count 60 ms toward the CSEs because it is very likely that the TC cell will generate a bad response. Thus, we try to identify the part of each silent episode during which bad TC responses happens due to post-inhibitory rebound. The above algorithm for calculating CSE is based on empirical observation in our data sets and numerical simulations. The fraction of CSE of the simulation time is defined as the contributed silent time (CST).

### Averaged GPi synaptic input to TC

**Figure 1:**
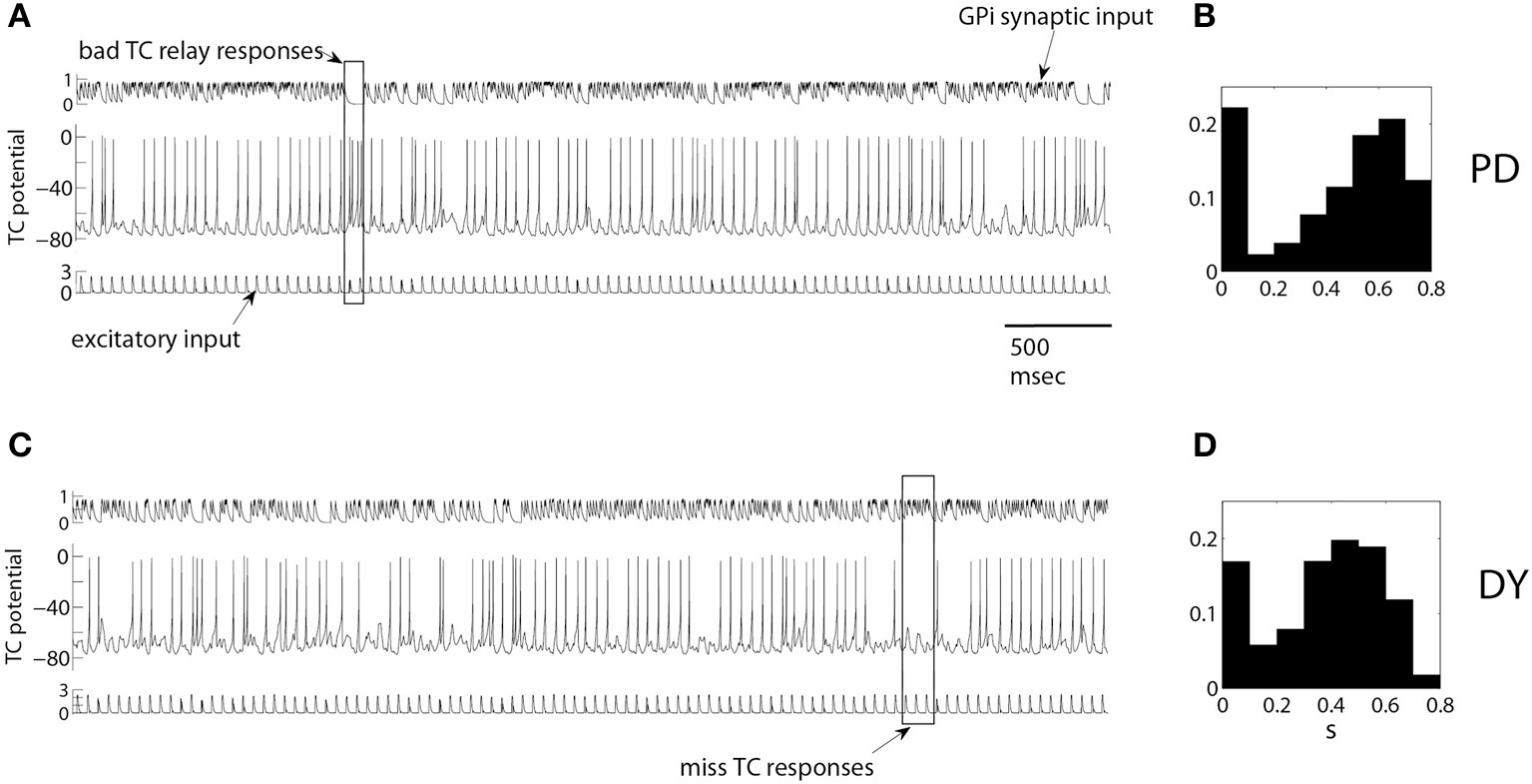
**Example of TC relay of periodic excitatory inputs to thalamus under pallidal inhibition in a parkinsonian (A,B) and dystonic (C,D) patients.** Upper traces in **(A,C)** are inhibitory synaptic input from GPi, middle traces are the activity of TC cells, bottom traces are excitatory synaptic input to TC cells. A rectangular box in **(A)** marks bad TC relay responses, rectangular box in **(C)** marks miss TC responses. **(B,D)** are the normalized histograms of the averaged synaptic input from pallidum to thalamus *s* as defined by Equation (3) (see *Averaged GPi synaptic input to TC* in *Methods* for the details). They show the probability that the value of *s* falls into the bins centered from 0.1 up to 0.8 with 0.1 increments. Synaptic input is high during elevated spiking episodes and low in between them, so that bimodal distribution points to the presence of bursting-like activity. The histograms are for one full episode of recording data from these patients.

Another good indicator of GPi rhythmicity is the variability of *s* averaged over a time window, which we call averaged GPi synaptic input. Based on (3), *s* is between 0 and 1. We choose 25 ms as our average time window. Then we take values for bin centers from 0.1 to 0.8 with 0.1 increments. We form histograms by displaying bins centered at 0.1–0.8 based on the frequency of the averaged *s* time course. If the average *s* value mostly falls into bin 0.1 and a bin away from bin 0.1, for example bin 0.6 in the top histogram displayed in the right panel of Figure 1, it shows that GPi firing is rhythmic with many ESEs. This is because GPi synaptic output is high during each ESEs and low between ESEs. Since we only have pathological GPi data recorded from patients, most of our data show high GPi rhythmicity.

Data analysis was performed in MATLAB. Model simulation was carried out with XPPAUT (http://www.math.pitt.edu/~bard/xpp/xpp.html).

## Results

### Examples of TC relay in DY and PD

We will start with presenting examples of TC relay of excitatory inputs (Figure 1) under inhibitory modulation of the thalamic cell by experimentally recorded GPi activity. We use a single GPi spike train in our simulation. Figures 1A,B presents the relay for a parkinsonian patient, Figures 1C,D presents relay for dystonia patient. GPi synaptic input (Figures 1A,C upper traces) was computed from experimentally recorded GPi activity as described in Methods. Figure 1 presents the membrane potential of TC relay cell responding to 5 s of excitatory inputs (Figures 1A,C lower traces) under modulation by such GPi synaptic input. Characteristic examples of miss and bad relay are indicated by rectangles.

The rectangular box in Figure 1A is an example of bad TC responses with more than one TC spike to one excitatory input. Synaptic input from pallidum is small here and TC cell shows a transient elevation of the firing rate, even though the excitatory input is still the same. These dynamics of the TC cell may be attributed to the rebound properties of TC cells due to T-type calcium current. The rectangular box in Figure 1C is an example of miss TC responses: several incoming excitatory spikes yield no spikes in the TC somatic activity, thus no spikes are relayed. The consistently high level of pallidal inhibition to TC during this time is apparently responsible for the miss responses.

The properties of the GPi modulatory inhibitory input in these data episodes are summarized by the histograms of the averaged GPi synaptic input, computed from the whole duration of the intraoperative recording episode (26.3 s of recordings for dystonia patient, Figure 1B; 41.8 s of recordings for parkinsonian patient, Figure 1D). Both histograms show rhythmicity in the GPi signal since the averaged GPi synaptic input, *s*, falls mostly in the lower bin 0.1 and in a bin (or a couple of bins) centered at a higher value. For example, in Figure 1B, the frequency of *s* as described in section *Averaged GPi synaptic input to TC*, in bin centered at 0.1 is high. And the frequencies of *s* in bins centered at 0.6 and 0.7 are high also. This shows that there are many ESEs and CSEs in the GPi signal, signifying the complex bursting nature of GPi activity.

These events of fidelity loss in TC relay and the underlying properties of GPi activity patterns were studied across our sample of parkinsonian and dystonic patients. We present the findings below.

### TC relay performance in PD and DY patients

The miss and bad indices of TC relay were computed using the data-driven computational model as described in the Methods for every patient in our sample of PD and DY patients. The values of the indices were computed for each episode under analysis. At the same time, the values for EST and CST, characteristics of the bursty discharge, were computed for GPi activity, so that one can inspect their relation to the miss and bad indices. This was done for both modeling setups: with regular and with random excitatory signals coming to the thalamocortical relay cell.

#### Parkinsonian patients

**Figure 2:**
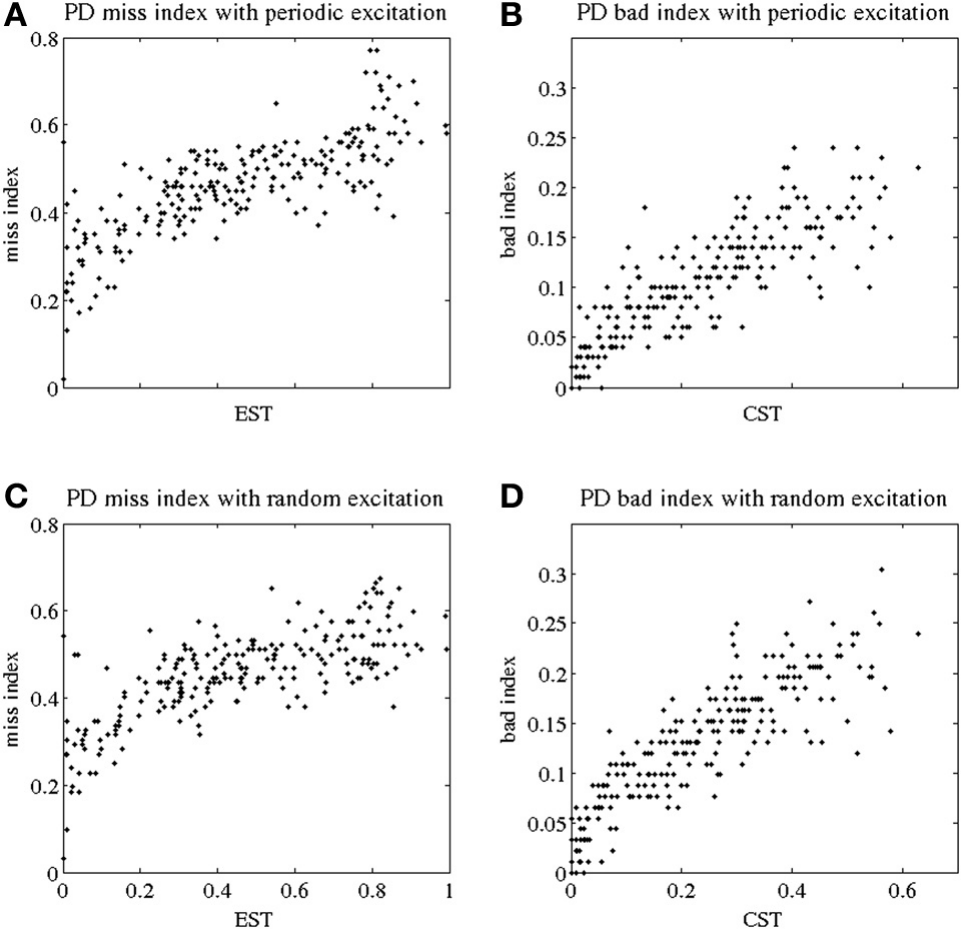
**Indices of fidelity of thalamocortical relay for parkinsonian patients.** Indices for non-transmitted spikes (miss indices) are at subplots **(A,C)**; indices for extra spikes (bad indices) are at subplots **(B,D)**. Periodic excitatory spike train to thalamocortical relay cell are at **(A,B)**; random excitation to thalamocortical relay cell are at **(C,D)**. The indices are plotted against EST (elevated spike time, the fraction of elevated spiking episodes over the total simulation time) and CST (contributed silent time, the fraction of all contributed silent episodes during the simulation time), see Methods.

The TC fidelity indices for parkinsonian patients are presented in Figure 2. In Figures 2A,C, we plot the miss index of TC relay against EST to both periodic and random excitatory inputs. (EST, the elevated spike time, was described in the subsection *GPi Elevated Spiking Time and CST*.) Each dot represents the miss index of TC relay to 5 s of GPi data recorded and derived from parkinsonian patients. The miss index spans an interval from 0 to 0.8 in both periodic and random excitation cases. The overall trend for miss index is to be positively (possibly linearly) correlated with EST. Figures 2B,D show the bad index of TC relay vs. CST (the CST, see subsection *GPi Elevated Spiking Time and CST*.) Figure 2B is TC relay bad index to periodic excitation, and Figure 2D is TC relay to random excitation. The bad index spans an interval from 0 to 0.3. The overall trend also shows positive relation between the bad index and CST. In Figure 2, we used all the parkinsonian patient data available to us.

Note that although the values of the thalamocortical relay error indices observed here are comparable with those reported in the primate study (Guo et al., 2008), one should compare them with a caution. First, we no longer use a single composite error index in this paper to characterize TC relay responses, but consider missed and bad responses separately. Second, in contrast to the earlier study of MPTP monkeys, here we consider human data. Third, some parameters of data analysis are different here. Finally, (Guo et al., 2008) presented a much smaller number of data points.

#### Dystonic patients

**Figure 3:**
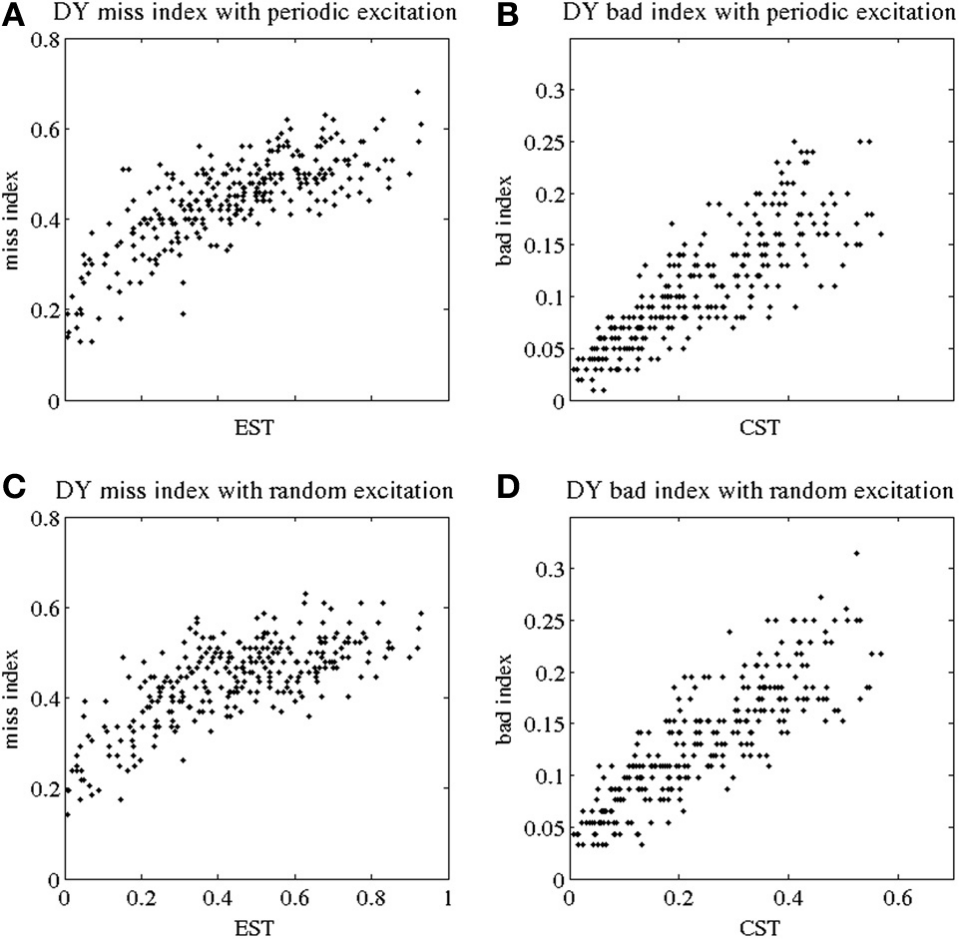
**Indices of fidelity of thalamocortical relay for dystonic patients.** Indices for non-transmitted spikes (miss indices) are at subplots **(A,C)**; indices for extra spikes (bad indices) are at subplots **(B,D)**. Periodic excitatory spike train to thalamocortical relay cell are at **(A,B)**; random excitation to thalamocortical relay cell are at **(C,D)**. The indices are plotted against EST (elevated spike time, the fraction of elevated spiking episodes over the total simulation time) and CST (contributed silent time, the fraction of all contributed silent episodes during the simulation time), see Methods.

Figures 3A,C show the miss index of TC relay vs. EST to both periodic and random excitatory inputs. Again each black dot gives the miss index of TC relay to 5 s of GPi data recorded and derived from dystonic patients. The miss index spans an interval from 0 to below 0.8 in both periodic and random excitation. Figures 3B,D show the bad index of TC relay vs. CST. Figure 3B is TC relay bad index to periodic excitation, and Figure 3D is TC relay to random excitation. Bad index spans values from 0 to 0.3. The overall trend of both miss and bad indices for dystonic patient data again shows a positive, possibly linear, relationship with EST and CST, respectively. Figure 3 includes the TC relay to all the dystonic patient data we have.

The TC relay in both parkinsonian and dystonic cases appears to be similar. The miss index in both cases spans an interval from 0 to 0.8, and the bad index in both is in the range from 0 to 0.3. The overall trend of both indices in parkinsonian and dystonic cases is the same. In addition, we performed a statistical analysis to explore the similarity. The results for the indices in both conditions is presented below.

#### Pallidal synaptic input to thalamus in parkinsonian and dystonic patients

**Figure 4:**
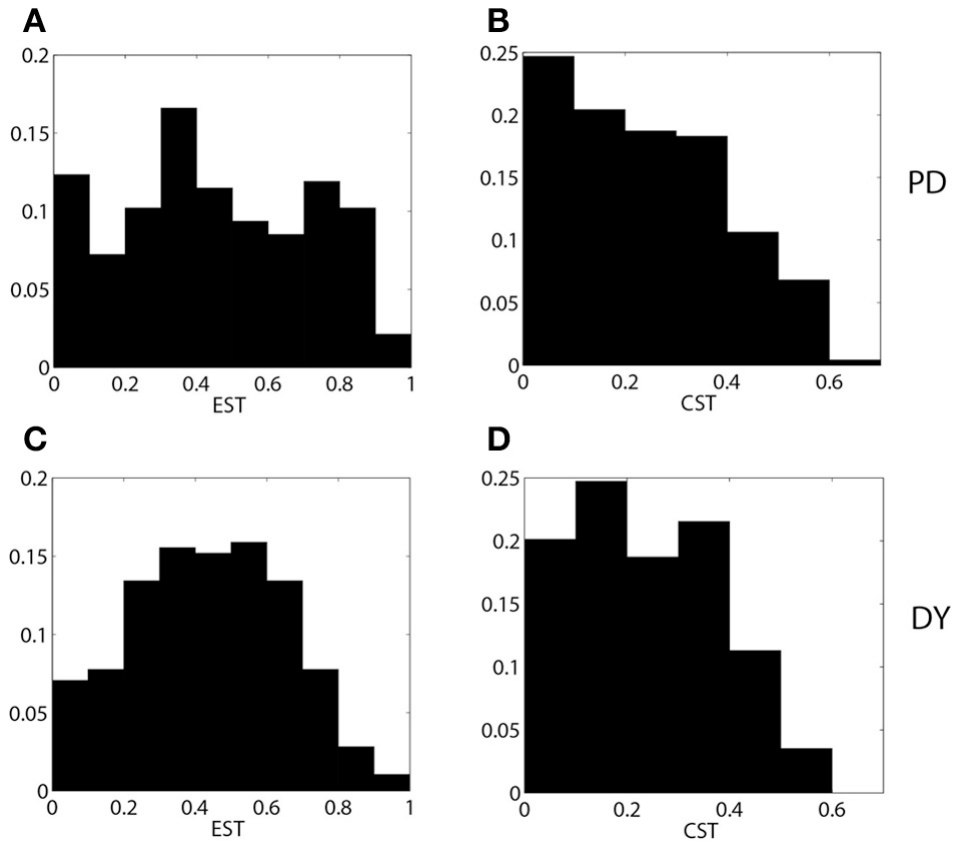
**The distributions of EST (A,C) and CST (B,D) for parkinsonian (A,B) and dystonic (C,D) data**.

**Figure 5:**
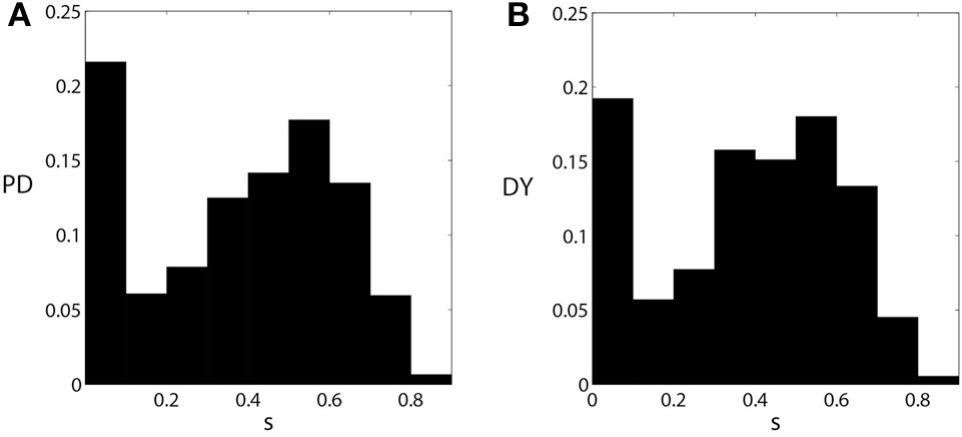
**The distributions of the averaged synaptic input from pallidum to thalamus s [as defined by Equation (3)] for parkinsonian (A) and dystonic (B) data.** These figures are similar to Figures 1B,D, but are computed from all the data used in this study.

We now compare the properties of pallidal activity and pallidal synaptic input to thalamus in parkinsonian and dystonic patients. The EST and CST distributions for PD and DY data are presented in Figure 4. The mean value of EST in PD (0.46) is very close to the mean value of EST in DY (0.44). However, the shapes of distributions appear to be different with a more pronounced peak in the case of dystonia. A similar effect is observed for the means of CST; both means are about 0.24, but the distributions appear to have different shape. The different shape of distributions should not be very surprising: the data come from different disorders noted for different kind of rhythmic neural activity. However, different distributions may lead to similar effects downstream. Interestingly, the distribution of the average GPi synaptic input variable, *s*, for dystonic and parkinsonian data are quite similar to each other (Figure 5). Thus, while the properties of pallidal neural activity (as measured by EST and CST) appear to be related to the indices of relay quality (Figures 2, 3), synaptic filtering at subthalamo-pallidal synapses may decrease the differences between dystonic and parkinsonian activity and contribute to the similarity of TC relay in both conditions.

### Statistical analysis

**Figure 6:**
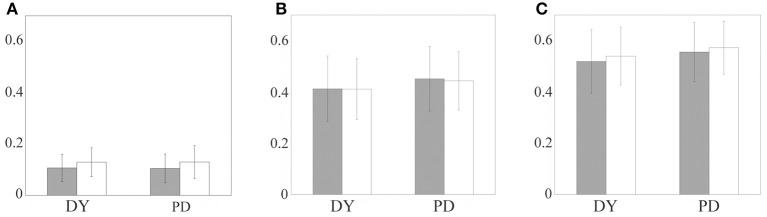
**The relay error indices for parkinsonian and dystonic patients: bad index, the fraction of additional spikes (A), miss index, the fraction of non-transmitted spikes (B), and error index, the sum of the first two (C).** The bars indicate mean values, the lines indicate standard deviations. Gray bars indicate periodic excitatory input, white bars indicate random excitatory input. Left pair of bars in each subplot is obtained from dystonic patients data, right pair of bars come from parkinsonian data.

We present mean values and standard deviations for our samples of miss index, bad index and joint error index in both regular and random excitation cases in Figure 6. First, one can see that the difference between the two modeling setups (periodic and random excitation) is very small for the miss index and error index (which is dominated mostly by the miss index), and is relatively small for the bad index. The results for regular and random excitation are not expected to be identical and the real excitatory input may be neither perfectly periodic nor random. However, in both cases, the values of the indices are similar, which points to the robust character of the modeling.

The difference in thalamocortical relay between parkinsonian and dystonic data is small also. Roughly speaking, the bad index is around 0.1, the miss index is around 0.4 and the error index is around 0.5 for all the cases considered. We applied the *t*-test to compare the means of distributions of the indices between dystonic and parkinsonian patients for each excitation type and for each index (i.e., comparison of gray bar to gray bar and white bar to white bar in each of the subplots of Figure 6). The bad index mean values for dystonia and Parkinson's disease are not significantly different at *p* = 0.01 (for both periodic and random excitations). The miss and error indices mean values for dystonia and Parkinson's disease are statistically significantly different at *p* = 0.01 (for both types of excitations). However, statistical significance does not imply the difference is large or functionally significant. Even though the *t*-test demonstrates a difference, the value of this difference is very small (see Figures 6B,C), i.e., much smaller than the values of the indices and their standard deviations.

For example, the 95% confidence interval for the difference of means of DY and PD miss index is (0.0125, 0.0515). The values of the means are 0.4107 and 0.4426. Thus, even the largest boundary of this interval is less than 13% of the smallest of the indices. The difference of means is less than 7.5% of the average of the means. Even more important is the observation that the difference between means is much smaller than the variance of either of the samples, PD and DY. More specifically, for the bad index, for periodic excitation the difference of means was −0.0019 (PD-DY) and the standard deviations were 0.056 (PD) and 0.053 (DY), for random excitation the difference of means was 0.0008 (PD-DY) and the standard deviations were 0.064 (PD) and 0.056 (DY). For the miss index, for periodic excitation the difference of means was 0.039 (PD-DY) and the standard deviations were 0.13 (PD) and 0.13 (DY), for random excitation the difference of means was 0.032 (PD-DY) and the standard deviations were 0.11 (PD) and 0.12 (DY). For the error index, for periodic excitation the difference of means was 0.037 (PD-DY) and the standard deviations were 0.12 (PD) and 0.12 (DY), for random excitation the difference of means was 0.035 (PD-DY) and the standard deviations were 0.10 (PD) and 0.11 (DY). Thus, the difference of means for bad index in PD and DY (Figure 6C) is more than an order of magnitude smaller than the standard deviations of error indices. The difference of means for miss index and error index are more than three times smaller than the standard deviations of these indices.

## Discussion

### Summary of findings

We studied TC relay response to excitatory inputs under the influence of GPi input in DY and PD patients using a data-driven computational model. TC is represented by a conductance-based model; excitatory inputs are computer-generated tonic inputs; the inhibitory modulation of TC by pallidum is obtained from experimentally recorded data from parkinsonian and dystonic patients. We observe that in both conditions, PD and DY, the modulation of TC by inhibitory pallidal input leads to infidelity of thalamocortical relay. That is, some of the excitatory spikes arriving at TC fail to elicit TC spikes, while sometimes TC generates a spike without any excitatory input. The amount of “missed” spikes and “bad” spikes (generated without input) is quantified by us in relation to the relayed spikes, yielding measures of relay quality.

We compared the relay quality indices for TC relay modulated by PD and DY pallidal activity. Both “miss” and “bad” indices (as well as their sum, “error” index) are very close to each other. The statistical tests are able to distinguish between the samples of indices in PD and DY, however, this difference is very small and is likely to be insignificant functionally. Statistics can distinguish between sufficiently large samples of two very similar distributions, which are virtually the same for most practical purposes. In the case considered here the difference between the mean values of all indices is more than an order of magnitude smaller than the mean values themselves. Even more importantly, the variances of indices in DY and PD largely overlap. In addition, the effect of random vs. regular excitation to TC generates small differences in indices which are comparable or larger than the differences in PD vs. DY. Thus, it is very reasonable to suggest that the differences in the quality of thalamocortical relay between dystonia and Parkinson's disease are very small and are likely to be functionally inconsequential.

It is interesting to note that the “bad” index is four times smaller than “miss” index. Apparently under inhibitory pallidal modulation TC is much more likely to miss a spike in response to an excitatory signal rather than to generate an extra spike. However, we would like to reiterate again that while the “bad” index is smaller than “mixed” index, the difference in each index between PD and DY is virtually non-existent.

### Robustness and potential pitfalls of the modeling approach

Any computational modeling study unavoidably omits (potentially important) details of the real experimental system and this study is no exception. There are several potential problems with the modeling which we would like to discuss here, as well as several considerations for why the overall conclusions may nevertheless be correct.

Dystonia is a heterogeneous disorder with many different subtypes which may have different etiologies and different electrophysiologies (Defazio et al., 2007; van der Salm et al., 2009). In this study, there was no selection bias, but the number of patients is relatively limited. This may diminish the relevance of the observations. However, the modeling results are robust and the outcomes for the different patients are similar to each other. So even though dystonia (and Parkinson's disease) may be inhomogeneous conditions, the resulting similarities of the thalamocortical relay imprecision are quite robust, which may suggest that there is a large population of dystonic and parkinsonian cases to which these results apply.

The two different kinds of excitatory stimuli for the TC cell do not lead to substantial differences in the outcome. Thus, the overall computational model and considered phenomenon are robust. Moreover, the switch from periodic to random excitation of TC generates larger differences in TC fidelity indices, than does the switch from PD to DY pallidal activity. This is an additional indication that TC fidelity is very similar in Parkinson's disease and dystonia.

The infidelity of thalamocortical relay is apparently caused by the bursty nature of the pallidal modulation of the thalamus. Bursting is hard to quantify by a single index. However, different degrees of burstiness may potentially lead to similar consequences downstream. Our results overcome this problem, because at the end we look at the thalamocortical relay quality with real experimentally recorded data, which does not depend on the index used to characterize the bursting.

While pallidal recordings from healthy humans are obviously not available, the data from normal monkeys provide a general idea for the values of the indices we observe here in a healthy state (Guo et al., 2008). The reported values of error index in MPTP parkinsonian rhesus macaques are around 0.6 and are similar to the values observed here in parkinsonian patients (about 0.5), while the error index values for the healthy primates are about two times smaller. It is reasonable to suggest that as in the PD state, the error index values in the healthy state are similar in monkeys and humans. Thus, the relatively high values of error indices we observe here in parkinsonian and dystonic humans are likely to be much larger than these indices in healthy humans. Thus, not only is the fidelity of thalamocortical relay similar in PD and DY, this fidelity is likely to be substantially smaller than that of healthy humans.

### Implications for dystonic and parkinsonian physiology

Complete understanding of the relationship between the TC relay properties and the physiology of both disorders is not possible before TC relay mechanisms and properties are well-understood and PD and DY symptomatic mechanisms are better characterized. However, by combining modeling and experimental data, our manuscript provides some interesting implications for potential PD and DY pathophysiological mechanisms. Our analysis shows no substantial differences in TC relay between the two conditions. As noted above, comparison with the healthy and MPTP monkeys strongly indicates that TC relay is substantially impaired in PD and thus in DY. While disruption of thalamic processing in PD was discussed earlier, our results suggest that a disruption of thalamic processing could also be involved in dystonia. Moreover, the degree to which TC relay fidelity is impaired is approximately the same in both conditions. While PD and DY may have much different pathologies and differ in oscillatory content of neural discharge (e.g., Starr et al., 2005; Crowell et al., 2012; Weinberger et al., 2012), our results indicate that the effect of patterning of pallidal discharge (which may elicit rebound bursts in thalamus preventing TC from relaying excitatory input, Rubin and Terman, 2004; Guo et al., 2008; Agarwal and Sarma, 2012) is similar in both conditions.

Since PD patients often present some dystonic symptomatology (Jankovic, 2008), it is not surprising that our results demonstrate similar effects on TC relay fidelity in the two conditions. This study does not explore correlations of particular features of TC relay with specific sets of motor symptoms. However, it suggests that even if the specifics of pallidal activity are different between PD and DY, the downstream effect on TC relay fidelity is the same in both conditions.

Furthermore, these results suggest that the mechanisms of GPi DBS in DY may involve improvement of fidelity of TC relay. It has previously been suggested that one of the ways in which high-frequency DBS of basal ganglia structures may improve motor behavior is via improvement of thalamocortical relay (Rubin and Terman, 2004; Guo et al., 2008; Guo and Rubin, 2011). DBS may regularize pallidal output which leads to a more efficient TC relay. Interestingly, experiments with DBS in dystonic hamsters indicate that DBS suppresses oscillatory power in a relatively broad frequency range, which extends from low frequencies to the higher frequencies, those in the beta band (Leblois et al., 2010), thus providing another evidence of a potential overlap of DBS action in dystonia and Parkinson's disease. Similarly, pallidotomy may partially remove bursty pallidal input to thalamus, improving fidelity of TC relay. As we observe here, TC relay in DY is similar to that in PD. Therefore, one of the potential mechanisms of pallidal DBS (or pallidotomy) in DY may be improvement of TC relay similar to that seen in PD. However, we should note that there are likely to be other mechanisms as well, because post-surgical improvement of symptoms in dystonia often demonstrates a delay in onset as compared to the improvement of motor symptoms in Parkinson's disease, which itself may follow different time courses for different symptoms (Lozano et al., 1997; Vidailhet et al., 2005, 2007).

The differences in the time-course of DBS improvements may be partially mediated by the substantial involvement of abnormalities of plasticity of sensorimotor circuitry in DY (Breakefield et al., 2008; Tamura et al., 2009; Peterson et al., 2010). Yet, when the stimulation is turned off, the symptoms return quickly (Vidailhet et al., 2005, 2007). So in spite of many differences in DBS and ablative surgeries in PD and DY with respect to the efficiency, target and time-course, there may be a common effect on TC relay.

Finally we would like to note that our observation of equally poor thalamocortical relay in both conditions suggests that while the basal ganglia is involved in both DY and PD, the differences in symptoms may be grounded in the pathophysiology of structures outside the basal ganglia (such as abnormalities of cortical and striatal plasticity, Breakefield et al., 2008; Tamura et al., 2009; Peterson et al., 2010) or in circuits other than the traditional motor circuitry implied in PD (such as potential disorganization of the oscillatory activity in the sensory domain in dystonia, Liu et al., 2008).

### Conflict of interest statement

The authors declare that the research was conducted in the absence of any commercial or financial relationships that could be construed as a potential conflict of interest.
